# Supplementary material for: Determinants and consequences of heavy episodic drinking among female sex workers in Ethiopia: A respondent-driven sampling study
Source: PLoS One. 2021 May 28;16(5):e0252432. doi: 10.1371/journal.pone.0252432 (PMC8162625; doi:10.1371/journal.pone.0252432)
Supplement: S1 Questionnaire — (DOCX) [file pone.0252432.s001.docx]

| **FSW INTERVIEW**  **SECTION 1**. **SCREENING INTERVIEW** | | | | | | | | | | | | | | | |  | | |  | |  |
| --- | --- | --- | --- | --- | --- | --- | --- | --- | --- | --- | --- | --- | --- | --- | --- | --- | --- | --- | --- | --- | --- |
| **Q.N** | | | **Questions and filters** | | | | | | | | | **Coding & response categories** | | | | **skip to** | | |  | |  |
| 10101 | | | RESPONDENT   ID | | | | | | | | | Site ID \|__\|__\| | | | |  | | |  | |  |
|  | | |  | | | | | | | | | Study Type ID \|__\|__\| | | | |  | | |  | |  |
|  | | |  | | | | | | | | | Subject ID \|__\|__\|__\| | | | |  | | |  | |  |
|  |  |  | CONSENT | | | | | | | | | SIGNED 1  ACCEPT NOT SIGNED 2  REFUSED 3 | | | |  | | |  |  |  |
|  |  |  | ENTRY TYPE | | | | | | | | | INTERVIEW 1  LAB RESULT 2 | | | |  | | |  |  |  |
| 10102 | | | INTERVIEWER ID | | | | | | | | | \|__\|__\| | | | |  | | |  | |  |
|  | | |  | | | | | | | | |  | | | |  | | |  | |  |
| 10103 | | | LOCATION | | | | | | | | | **REGIONAL CAPITALS** | | | |  | | |  | |  |
|  | | |  | | | | | | | | | ADDIS ABABA 1 | | | |  | | |  | |  |
|  | | |  | | | | | | | | | BAHIR DAR 2 | | | |  | | |  | |  |
|  | | |  | | | | | | | | | HAWASSA 3 | | | |  | | |  | |  |
|  | | |  | | | | | | | | | MAKELE 4 | | | |  | | |  | |  |
|  | | |  | | | | | | | | | GAMBELLA 5 | | | |  | | |  | |  |
|  | | |  | | | | | | | | | ADAMA 6 | | | |  | | |  | |  |
|  | | |  | | | | | | | | | **TRANSPORT CORRIDORS** | | | |  | | |  | |  |
|  | | |  | | | | | | | | | KOMBOLCHA 7 | | | |  | | |  | |  |
|  | | |  | | | | | | | | | SAMERA-LOGIA 8 | | | |  | | |  | |  |
|  | | |  | | | | | | | | | METEMA 9 | | | |  | | |  | |  |
|  | | |  | | | | | | | | | SHASHEMENE 10  DIRE DAWA 11 | | | |  | | |  | |  |
|  | | |  | | | | | | | | |  | | | |  | | |  | |  |
| 10104 | | | DATE (Ethiopian) | | | | | | | | | ___ ___/ ___ ___ / ___ ___ ___ ___ | | | |  | | |  | |  |
|  | | |  | | | | | | | | | D D M M Y Y | | | |  | | |  | |  |
| 10105 | | | TIME(24 hours) started | | | | | | | | | ___ ___ : ___ ___ | | | |  | | |  | |  |
|  | | | System Time started | | | | | | | | | ___ ___ : ___ ___ | | | |  | | |  | |  |
|  | | |  | | | | | | | | | H H M M | | | |  | | |  | |  |
|  | | | **Coupon number** | | | | | | | | | _________________________________ | | | |  | | |  | |  |
|  | | | **Eligibility Criteria** | | | | | | | | |  | | | |  | | |  | |  |
| 10106 | | | Have you been living in this town for at least the last 30 days? | | | | | | | | | YES 1 | | | |  | | |  | |  |
|  | | |  |  |  |  |  |  |  |  |  | NO 2 | | | | Ineligible | | |  | |  |
| 10107 | | | Have you exchanged sex for money or for other benefit in this town in the last 30 days? | | | | | | | | | YES 1 | | | |  | | |  | |  |
|  | | |  |  |  |  |  |  |  |  |  | NO 2 | | | | Ineligible | | |  | |  |
| 10108 | | | How many different men have you sold sex in this town in the last 7 days? | | | | | | | | | \|__\|__\|__\|  **if no register 000** | | | |  | | |  | |  |
| 10109 | | | How many different men have you sold sex in this town in the last 30 days? | | | | | | | | | \|__\|__\|__\| | | | |  | | |  | |  |
|  | | |  | | | | | | | | | **001 - 003** | | | | Ineligible | | |  | |  |
| 10110 | | | How old are you? | | | | | | | | | [__\|__] | | | |  | | |  | |  |
|  | | |  | | | | | | | | | **If age<15** | | | | Ineligible | | |  | |  |
|  | | | ***If the respondent is the seed*** | | | | | | | | | ***Skip to*** | | | | ***10114*** | | |  | |  |
|  | | |  | | | | | | | | |  | | | |  | | |  | |  |
| 10111 | | | Was the person who gave the coupon to you a woman or a man? | | | | | | | | | WOMAN 1 | | | |  | | |  | |  |
|  | | |  |  |  |  |  |  |  |  |  | MAN 2 | | | | Ineligible | | |  | |  |
| 10112 | | | Do you know this person by name or by sight*?* | | | | | | | | | YES 1 | | | |  | | |  | |  |
|  | | | ***PROBE: is this friend or acquaintance?*** | | | | | | | | | NO 2 | | | | Ineligible | | |  | |  |
| 10113 | | | How did you get this coupon? | | | | | | | | | FROM ANOTHER SEX WORKER 1 | | | |  | | |  | |  |
|  | | | ***Do not read the response category*** | | | | | | | | | FROM SOMEONE ELSE 2 | | | | Ineligible | | |  | |  |
|  | | |  | | | | | | | | | FOUND ON THE GROUND 3 | | | | Ineligible | | |  | |  |
|  | | |  |  |  |  |  |  |  |  |  | OTHERS 4 | | | | Ineligible | | |  | |  |
| 10114 | | | Before we started this survey we distributed a keychain. Did you receive a keychain?  *[****Make clear to respondent that this answer will not affect their participation]***  If yes, ask her to show it. | | | | | | | | | YES, I RECEIVED AND HAVE IT WITH  ME 1  YES, I RECEIVED BUT DO NOT HAVE IT WITH ME 2  NO, I DID NOT RECEIVE 3 | | | | 10116  10116 | | |  | |  |
| 10115 | | | If respondent says yes but did not bring it, show 5 sample key chains and ask her to pick out the correct one. | | | | | | | | | CHOOSES CORRECT KEYCHAIN 1  CHOOSES AN INCORRECT ONE 2  DOESN’T CHOOSE ANYONE 3 | | | |  | | |  | |  |
| 10116 | | | Are you a member of a _______ [Sex worker association] or ____ [peer education group] establish by _________ | | | | | | | | | YES 1  NO 2 | | | |  | | |  | |  |
|  | | | **Is the respondent** | | | | | | | | | Eligible 1  Ineligible 2 | | | |  | | |  | |  |
|  | | | **SECTION 1. NETWORK QUESTIONS** | | | | | | | | | | | | |  | | |  | |  |
| **I would like to first ask you some questions about other FSW that you may know. Please remember that no one will be able to find out what you tell me so please be truthful in your answers** | | | | | | | | | | | | | | | | | | |  | |  |
|  |  |  |  |  |  |  |  |  |  |  |  |  |  |  |  |  |  |  |  | |  |
|  |  |  |  |  |  |  |  |  |  |  |  |  |  |  |  |  |  |  |  | |  |
|  |  | | | | | | | | | | | |  | | | |  | |  | |  |
| **Q.N** | **Questions** | | | | | | | | | | | | **Coding & response categories** | | | | **Skip** | |  | |  |
|  | Think about the kind of people we are looking for in this survey (FSW in this city). Think about the people you know by name or sight who would be the right people to join the survey. | | | | | | | | | | | |  | | | |  | |  | |  |
|  |  |  |  |  |  |  |  |  |  |  |  |  |  | | | |  | |  | |  |
|  |  |  |  |  |  |  |  |  |  |  |  |  |  | | | |  | |  | |  |
|  |  |  |  |  |  |  |  |  |  |  |  |  |  | | | |  | |  | |  |
| 10117 | How many of these friends and acquaintances who are 15 years & above have you seen in the last **7** days?  **(Probe and make sure you get the recruit’s best guess**) | | | | | | | | | | | | AVERAGE NUMBER [__\|__\|__] | | | |  | |  | |  |
|  |  |  |  |  |  |  |  |  |  |  |  |  |  | | | |  | |  | |  |
| 10118 | How many of these friends and acquaintances who are 15 years & above have you seen in the last **30** days?  (**Probe and make sure you get the recruit’s best guess**) | | | | | | | | | | | | AVERAGE NUMBER [__\|__\|__] | | | |  | |  | |  |
|  |  |  |  |  |  |  |  |  |  |  |  |  |  | | | |  | |  | |  |
|  |  |  |  |  |  |  |  |  |  |  |  |  |  | | | |  | |  | |  |
|  |  | | | | | | | | | | | |  | | | |  | |  | |  |
| **SECTION 2.    BACKGROUND CHARACTERSTICS** | | | | | | | | | | | | | | | | | | | |  |  |
| **Q.N** | **Question** | | | | | | | | **Coding & response categories** | | | | | | **Remark** | | | |  | |  |
| 10201 | In which region were you born? | | | | | | | | AMHARA 1 | | | | | |  | | | |  | |  |
|  |  | | | | | | | | OROMIA 2 | | | | | |  | | | |  | |  |
|  |  | | | | | | | | SNNPR 3 | | | | | |  | | | |  | |  |
|  |  | | | | | | | | TIGRAI 4 | | | | | |  | | | |  | |  |
|  |  | | | | | | | | ADDIS ABABA 5 | | | | | |  | | | |  | |  |
|  |  | | | | | | | | DIRE DAWA 6 | | | | | |  | | | |  | |  |
|  |  | | | | | | | | HARAR 7 | | | | | |  | | | |  | |  |
|  |  | | | | | | | | AFAR 8 | | | | | |  | | | |  | |  |
|  |  | | | | | | | | SOMALI 9 | | | | | |  | | | |  | |  |
|  |  | | | | | | | | BENISH.-GUMUZ 10 | | | | | |  | | | |  | |  |
|  |  | | | | | | | | GAMBELA 11 | | | | | |  | | | |  | |  |
|  |  | | | | | | | | OUTSIDE ETHIOPIA 12 | | | | | |  | | | |  | |  |
| 10202 | What is your nationality? | | | | | | | | ETHIOPIAN 1 | | | | | |  | | | |  | |  |
|  |  | | | | | | | | ERITREAN 2 | | | | | |  | | | |  | |  |
|  |  | | | | | | | | SOMALIAN 3 | | | | | |  | | | |  | |  |
|  |  | | | | | | | | SUDANESE 4 | | | | | |  | | | |  | |  |
|  |  | | | | | | | | KENYAN 5 | | | | | |  | | | |  | |  |
|  |  | | | | | | | | DJIBUTI 6 | | | | | |  | | | |  | |  |
|  |  | | | | | | | | OTHER COUNTRY 7 | | | | | |  | | | |  | |  |
| 10203 | What is the highest grade of schooling you attained? | | | | | | | | No education 0 | | | | | |  | | | |  | |  |
|  |  |  |  |  |  |  |  |  | Non-formal education 1 | | | | | |  | | | |  | |  |
|  |  |  |  |  |  |  |  |  | Primary 1^st^ cycle (grade 1-4) 2  Primary 2^nd^ cycle (grade 5-8) 3 | | | | | |  | | | |  | |  |
|  |  |  |  |  |  |  |  |  | Secondary school (grade 9-10)4  Secondary preparatory (grade11-12)5  TVET 6  College diploma 7  Degree & above 8  NO RESPONSE 9 | | | | | |  | | | |  | |  |
|  |  | | | | | | | |  | | | | | |  | | | |  | |  |
| 10204 | At what age did you start sexual intercourse? | | | | | | | | AGE IN YEARS \|__\|__\|  DON’T KNOW 98  REFUSED TO ANSWER 99 | | | | | |  | | | |  |  |  |
| 10205 | What is your current marital status? | | | | | | | | NEVER MARRIED 1 | | | | | | Skip to 10208 | | | |  |  |  |
|  |  | | | | | | | | MARRIED 2 | | | | | |  | | | |  |  |  |
|  |  | | | | | | | | COHABITATION 3 | | | | | |  | | | |  | |  |
|  |  | | | | | | | | DIVORCED 4 | | | | | | 10207 | | | |  | |  |
|  |  | | | | | | | | SEPARATED 5 | | | | | | 10208 | | | |  | |  |
|  |  | | | | | | | | WIDOWED 6 | | | | | | 10207 | | | |  | |  |
|  |  | | | | | | | | REFUSED TO ANSWER 9 | | | | | | 10208 | | | |  | |  |
| 10206 | How old were you when you first married/began cohabiting? | | | | | | | |  | | | | | |  | | | |  | |  |
|  |  |  |  |  |  |  |  |  | \|__\|__\| AGE | | | | | |  | | | |  | |  |
|  | ***Probe*** | | | | | | | | DON’T KNOW 98 | | | | | |  | | | |  | |  |
|  |  | | | | | | | | REFUSED TO ANSWER 99 | | | | | |  | | | |  | |  |
| 10207 | How old were you when you first widowed /divorced? | | | | | | | |  | | | | | |  | | | |  | |  |
|  |  |  |  |  |  |  |  |  | \|___\|___\| AGE  I HAVE NEVER BEENWIDOWED/DIVORCED 97 | | | | | |  | | | |  | |  |
|  |  |  |  |  |  |  |  |  | DON’T KNOW 98 | | | | | |  | | | |  | |  |
|  |  | | | | | | | | REFUSED TO ANSWER 99 | | | | | |  | | | |  | |  |
| 10208 | Have you ever given birth? | | | | | | | | YES 1  NO 2 | | | | | | 10211 | | | |  | |  |
|  |  | | | | | | | |  | | | | | |  | | | |  | |  |
| 10209 | If Yes, How many of your children are surviving? ` | | | | | | | | \|__\|__\| | | | | | |  | | | |  | |  |
|  |  | | | | | | | | NONE 00 | | | | | | 10211 | | | |  | |  |
| 10210 | How many live together with you now? | | | | | | | | \|__\|__\| | | | | | |  | | | |  | |  |
|  |  | | | | | | | | NONE 00 | | | | | |  | | | |  | |  |
| 10211 | Are you currently using a modern contraceptive (besides condoms)? | | | | | | | | None 1  PILL 2 | | | | | |  | | | |  | |  |
|  |  |  |  |  |  |  |  |  | IUD 3 | | | | | |  | | | |  | |  |
|  |  |  |  |  |  |  |  |  | Inject able 4 | | | | | |  | | | |  | |  |
|  |  |  |  |  |  |  |  |  | Implant 5  Sterilization 6  Emergency contraceptives 7 | | | | | |  | | | |  | |  |
|  |  |  |  |  |  |  |  |  |  | | | | | |  | | | |  | |  |
| 10212 | Many sex workers became pregnant as the result of their work and have to abort. How many pregnancies like this have you terminated? | | | | | | | | \|__\|__\| | | | | | |  | | | |  | |  |
|  |  |  |  |  |  |  |  |  | NONE 00 | | | | | |  | | | |  | |  |
|  |  | | | | | | | |  | | | | | |  | | | |  | |  |
| **SECTION 3. TRANSITION TO SEXWORK** | | | | | | | | | | | | | | |  | | | |  | |  |
| **Q.N** | | **Questions** | | | | | | | | | **Coding & response categories** | | | | **Remark** | | | |  | |  |
| 10301 | | How old were you when you first started selling sex on a regular basis? | | | | | | | | | AGE IN COMPLETED YEARS \|___\|___\| | | | |  | | | |  | |  |
|  | |  |  |  |  |  |  |  |  |  |  | | | |  | | | |  | |  |
|  | |  | | | | | | | | | Don’t remember 97 | | | |  | | | |  | |  |
| 10302 | | What was your marital status when you first started selling on a regular basis? | | | | | | | | | NEVER MARRIED 1 | | | |  | | | |  | |  |
|  | |  |  |  |  |  |  |  |  |  | CURRENTLY MARRIED 2 | | | |  | | | |  | |  |
|  | |  |  |  |  |  |  |  |  |  | COHABITATION 3 | | | |  | | | |  | |  |
|  | |  |  |  |  |  |  |  |  |  | DIVORCED 4 | | | |  | | | |  | |  |
|  | |  |  |  |  |  |  |  |  |  | SEPARATED 5 | | | |  |  |  |  |  | |  |
|  | |  | | | | | | | | | WIDOWED 6 | | | |  | | | |  | |  |
|  | |  | | | | | | | | | REFUSED TO ANSWER 9 | | | |  |  |  |  |  | |  |
| 10303 | | Did you have a job before started selling sex? What was your job immediately before you started selling sex? | | | | | | | | | NONE 0 | | | |  | | | |  | |  |
|  | |  |  |  |  |  |  |  |  |  | HOUSE MAID 1 | | | |  | | | |  | |  |
|  | |  |  |  |  |  |  |  |  |  | BAR/HOTEL/ENTERTAINMENT 2 | | | |  | | | |  | |  |
|  | |  |  |  |  |  |  |  |  |  | RETAIL/TRADE 3 | | | |  | | | |  | |  |
|  | |  |  |  |  |  |  |  |  |  | FARMING/LIVESTOCK 4 | | | |  | | | |  | |  |
|  | |  |  |  |  |  |  |  |  |  | OTHER SKILLED MANUAL 5  OTHER SKILLED NON-MANUAL 6 | | | |  | | | |  | |  |
|  | |  | | | | | | | | | OTHER UNSKILLED MANUAL 7 | | | |  | | | |  | |  |
|  | |  | | | | | | | | | STUDENT 8 | | | |  | | | |  | |  |
|  | |  | | | | | | | | | HOUSE WIFE 9  DON’T KNOW 98  REFUSED TO ANSWER 99 | | | |  | | | |  | |  |
| 10304 | | Would you say someone forced you into selling sex? | | | | | | | | | NO ONE 0 | | | |  | | | |  | |  |
|  | |  |  |  |  |  |  |  |  |  | MIDDLE PERSON 1 | | | |  | | | |  | |  |
|  | |  |  |  |  |  |  |  |  |  | BOSS 2 | | | |  | | | |  | |  |
|  | |  |  |  |  |  |  |  |  |  | FRIENDS/CO-WORKERS 3 | | | |  | | | |  | |  |
|  | |  | | | | | | | | | SEXUAL PARTNER 4 | | | |  | | | |  | |  |
|  | |  | | | | | | | | | FAMILY MEMBERS 5 | | | |  | | | |  | |  |
|  | |  | | | | | | | | | OTHERS 6 | | | |  | | | |  | |  |
| 10305 | | Some people get cheated into sex work. Did this happen to you? If so, who cheated you? | | | | | | | | | NO ONE 0 | | | |  | | | |  | |  |
|  | |  |  |  |  |  |  |  |  |  | MIDDLE PERSON 1 | | | |  | | | |  | |  |
|  | |  |  |  |  |  |  |  |  |  | BOSS 2 | | | |  | | | |  | |  |
|  | |  | | | | | | | | | FRIENDS/CO-WORKERS 3 | | | |  | | | |  | |  |
|  | |  | | | | | | | | | SEXUAL PARTNER 4 | | | |  | | | |  | |  |
|  | |  | | | | | | | | | FAMILY MEMBERS 5  OTHERs 6  REFUSED TO RESPONSE 9 | | | |  | | | |  | |  |
| 10306 | | Was there anyone who influenced you to start selling sex? | | | | | | | | | NO ONE 0 | | | |  | | | |  | |  |
|  | |  |  |  |  |  |  |  |  |  | MIDDLE PERSON 1 | | | |  | | | |  | |  |
|  | |  |  |  |  |  |  |  |  |  | BOSS 2 | | | |  | | | |  | |  |
|  | |  |  |  |  |  |  |  |  |  | FRIENDS/CO-WORKERS 3 | | | |  | | | |  | |  |
|  | |  | | | | | | | | | SEXUAL PARTNER 4 | | | |  | | | |  | |  |
|  | |  | | | | | | | | | FAMILY MEMBERS 5 | | | |  | | | |  | |  |
|  | |  | | | | | | | | | OTHERs 6  REFUSED TO RESPONSE 9 | | | |  | | | |  | |  |
| 10307 | | Why did you start selling sex? | | | | | | | | | TO HELP MY FAMILIES/CHILDREN 1 | | | |  | | | |  | |  |
|  | |  |  |  |  |  |  |  |  |  | TO GET BETTER INCOME 2 | | | |  | | | |  | |  |
|  | |  | | | | | | | | | DUE TO MY PARENTS DEATH 3 | | | |  | | | |  | |  |
|  | | **Circle all mentioned** | | | | | | | | | DIVORCED WITH MY HUSBAND 4 | | | |  | | | |  | |  |
|  | |  | | | | | | | | | QUARREL WITH FAMILY 5 | | | |  | | | |  | |  |
|  | |  | | | | | | | | | NO OTHER OPTION OR JOB 6 | | | |  | | | |  | |  |
|  | |  | | | | | | | | | DUE TO DEBTS OF MONEY 7 | | | |  | | | |  | |  |
|  | |  | | | | | | | | |  | | | |  | | | |  | |  |
|  | |  | | | | | | | | |  | | | |  | | | |  | |  |
| **SECTION 4 MOBILITY AND CURRENT WORK** | | | | | | | | | | | | | | |  | | | |  | |  |
| **Q.N** | | | | **Questions** | | **Coding & response categories** | | | | | | | | | **Remark** | | | |  | |  |
| 10401 | | | | How long ago did you move to this city? | | 0 to 3 months  1  4 to 6 months 2  7 to 12 months 3  13 to 23 months 4  2 and above years 5 | | | | | | | | |  | | | |  | |  |
|  | | | |  |  | I WAS BORN HERE AND DID NOT MOVE 6 | | | | | | | | |  | | | |  | |  |
|  | | | |  |  |  | | | | | | | | |  | | | |  | |  |
|  | | | |  | |  | | | | | | | | |  | | | |  | |  |
|  | | | |  | |  | | | | | | | | |  | | | |  | |  |
| 10402 | | | | How many different cities or towns have you sold sex in the last 3 years? **(including current city/town)** | | \|__\|__\| | | | | | | | | |  | | | |  | |  |
|  | | | |  |  | DON’T REMEMBER 97 | | | | | | | | |  | | | |  | |  |
|  | | | |  |  |  | | | | | | | | |  | | | |  | |  |
| 10403 | | | | What types of areas have you sold sex in? READ LIST  Circle all mentioned | | ADDIS ABABA 1 | | | | | | | | |  | | | |  | |  |
|  |  |  |  |  |  | OTHER REGIONAL CAPITALS 2 | | | | | | | | |  | | | |  | |  |
|  |  |  |  |  |  | OTHER CITIES OR TOWNS 3 | | | | | | | | |  | | | |  | |  |
|  |  |  |  |  |  | CONSTRUCTION SITES 4  RURAL AREA 5 | | | | | | | | |  | | | |  | |  |
|  | | | |  | |  | | | | | | | | |  | | | |  | |  |
|  | | | | **CURRENT WORK ENVIRONMENT** | | | | | | | | | | | **Skip to** | | | |  | |  |
| 10404 | | | | How/Where do you usually meet your clients to sell sex? | | BAR/ HOTEL 0  Local drink house (Arakebet, Tellabet, Tejbet) 1 | | | | | | | | |  | | | |  | |  |
|  |  |  |  |  |  | RESTAURANT/CAFE /CAKE BET 2  SPA / MASSAGE / BEAUTY 3  OWN HOME 4 | | | | | | | | |  | | | |  | |  |
|  |  |  |  |  |  | RED LIGHT 5 | | | | | | | | | skip to 10406 | | | |  | |  |
|  |  |  |  |  |  | STREET 6 | | | | | | | | |  |  |  |  |  | |  |
|  |  |  |  |  |  | SMS/ PHONE 7 | | | | | | | | |  |  |  |  |  | |  |
|  | | | |  | | INTERNET 8 | | | | | | | | |  |  |  |  |  | |  |
|  | | | |  | | OTHER 9 | | | | | | | | |  |  |  |  |  | |  |
| 10405 | | | | Do you do any additional work there besides selling sex? | | YES 1 | | | | | | | | |  | | | |  | |  |
|  |  |  |  |  |  | NO 2 | | | | | | | | | Skip to 10407 | | | |  | |  |
| 10406 | | | | Do you do any other kind of regular work to earn money? If so what is your main job? | | NONE 1 | | | | | | | | |  | | | |  | |  |
|  |  |  |  |  |  | PAID HOUSE MAID 2 | | | | | | | | |  | | | |  | |  |
|  |  |  |  |  |  | BAR/HOTEL/ENTERTAINMENT 3 | | | | | | | | |  | | | |  | |  |
|  |  |  |  |  |  | RETAIL/TRADE 4 | | | | | | | | |  | | | |  | |  |
|  |  |  |  |  |  | FARMING/LIVESTOCK 5 | | | | | | | | |  | | | |  | |  |
|  |  |  |  |  | | OTHER SKILLED MANUAL 6 | | | | | | | | |  | | | |  | |  |
|  | | | |  | | OTHER SKILLED NON-MANUAL 7  OTHER UNSKILLED MANUAL 8 | | | | | | | | |  | | | |  | |  |
| 10407 | | | | How much do you earn on average in a month from selling sex? | | \|__\|__\|__\|__\|__\| ETB | | | | | | | | |  | | | |  | |  |
| 10408 | | | | How much money do you earn in average in a month from your other sources besides selling sex? | | \|__\|__\|__\|__\|__\| ETB | | | | | | | | |  | | | |  | |  |
| 10409 | | | | Are you obliged to share income from sex with anyone? With mostly who? | | NO ONE 1 | | | | | | | | |  | | | |  | |  |
|  |  |  |  |  |  | OWNER OF WORKPLACE 2 | | | | | | | | |  | | | |  | |  |
|  |  |  |  |  |  | MIDDLE PERSON 3 | | | | | | | | |  | | | |  | |  |
|  |  |  |  |  | | REGULAR SEXUAL PARTNER 4 | | | | | | | | |  | | | |  | |  |
|  |  |  |  |  | | FAMILY 5 | | | | | | | | |  | | | |  | |  |
|  | | | |  | | OTHERS 6 | | | | | | | | |  | | | |  | |  |
|  | | | |  | |  | | | | | | | | |  | | | |  | |  |
| 10410 | | | | Do you provide any regular financial or other support to anyone (including children)? Who do you support? PROBE: Anyone else?  ***CIRCLE ALL MENTIONED*** | | NO ONE 1 | | | | | | | | | 10411 | | | |  | |  |
|  |  |  |  |  |  | CHILDREN 2 | | | | | | | | |  | | | |  | |  |
|  |  |  |  |  |  | PARENTS 3 | | | | | | | | |  | | | |  | |  |
|  |  |  |  |  |  | SIBLINGS 4 | | | | | | | | |  | | | |  | |  |
|  |  |  |  |  |  | OTHER RELATIVES 5 | | | | | | | | |  | | | |  | |  |
|  |  |  |  |  |  | SPOUSE 6 | | | | | | | | |  | | | |  | |  |
|  |  |  |  |  |  | FRIENDS 7 | | | | | | | | |  | | | |  | |  |
|  | | | |  | | OTHERS 8 | | | | | | | | |  | | | |  | |  |
| 10411 | | | | Do you save money regularly? If yes how do you save money? | | I DON’T SAVE 1  KEPT IN BANK 2 | | | | | | | | |  | | | |  | |  |
|  |  |  |  |  |  | EQUB3 | | | | | | | | |  | | | |  | |  |
|  |  |  |  |  |  | SAVING & CREDIT ENTERPRISE 4  KEEP AT HOME 5  KEEPING WITH RELATIVES/FRIENDS/BOSS 6  OTHER 7 | | | | | | | | |  | | | |  | |  |
|  | | | |  | |  | | | | | | | | |  | | | |  | |  |
| 10412 | | | | Do you currently have any debts that will take you more than one month to repay? | | YES 1 | | | | | | | | |  | | | |  | |  |
|  |  |  |  |  |  | NO 2 | | | | | | | | |  | | | |  | |  |
|  |  |  |  |  |  | REFUSED TO ANSWER 9 | | | | | | | | |  | | | |  | |  |
|  | | | |  | | | | | | | | | |  |  | | | |  | |  |
| **SECTION 5. EXPOSURE TO SEXUAL RISK : GENERAL** | | | | | | | | | | | | | | |  | | | |  | |  |
| **Q.N** | | **Questions** | | | | | |  | | **Coding & response categories** | | | | | **Skip to** | | |  | | | |
|  | | **LAST PAYING PARTNER** | | | | | |  | | | | | | |  | | | |  | |  |
|  | | Think of the most recent paying sexual partner you had: | | | | | | | | | | | | |  | | | |  | |  |
| 10501 | | What is your last paying partner’s occupation? | | | | UNEMPLOYED 0 | | | | | | | | |  | | | |  | |  |
|  | |  |  |  |  | CIVIL SERVANT 1 | | | | | | | | |  | | | |  | |  |
|  | |  |  |  |  | TRANSPORT WORKER 2 | | | | | | | | |  | | | |  | |  |
|  | |  | | | | CONSTRUCTION WORKER/DAILY LABORER 3 | | | | | | | | |  | | | |  | |  |
|  | |  | | | | STUDENT 4 | | | | | | | | |  | | | |  | |  |
|  | |  | | | | FARMER 5 | | | | | | | | |  | | | |  | |  |
|  | |  | | | | UNIFORMED SERVICE 6 | | | | | | | | |  | | | |  | |  |
|  | |  | | | | BUSINESS/TRADE/RETAIL 7 | | | | | | | | |  | | | |  | |  |
|  | |  | | | | OTHERS( NOT MENTIONED ABOVE) 8  DON’T KNOW 9 | | | | | | | | |  | | | |  | |  |
| 10502 | | The last time you had sex with this partner, had the partner been drinking? A little or a lot? | | | | NOT DRINKING 1 | | | | | | | | |  | | | |  | |  |
|  | |  |  |  |  | DRINKING A LITTLE 2 | | | | | | | | |  | | | |  | |  |
|  | |  |  |  |  | DRINKING A LOT 3 | | | | | | | | |  | | | |  | |  |
|  | |  |  |  |  | DON’T KNOW 8 | | | | | | | | |  | | | |  | |  |
| 10503 | | The last time you had sex with this partner, had you been drinking? A little or a lot? | | | | NOT DRINKING 1 | | | | | | | | |  | | | |  | |  |
|  | |  |  |  |  | DRINKING A LITTLE 2 | | | | | | | | |  | | | |  | |  |
|  | |  |  |  |  | DRINKING A LOT 3  DON’T KNOW 8 | | | | | | | | |  | | | |  | |  |
| 10504 | | Did you use condom in the last sexual intercourse? | | | | YES 1 | | | | | | | | | 10506 | | | |  | |  |
|  | |  |  |  |  | NO 2 | | | | | | | | |  | | | |  | |  |
|  | |  |  |  |  | DON’T REMEMBER 7 | | | | | | | | | 10506 | | | |  | |  |
|  | | Why didn’t you use condom? | | | | CONDOM WAS NOT AVAILABLE 1  PAID ME MORE TO NOT USE A CONDOM 2 | | | | | | | | |  | | | |  | |  |
| 10505 | |  |  |  |  | TOO EXPENSIVE 3 | | | | | | | | |  | | | |  | |  |
|  | |  | | | | PARTNER OBJECTION 4 | | | | | | | | |  | | | |  | |  |
|  | |  | | | | USED OTHER CONTRACEPTIVE 5  FORCED BY PARTNER 6 | | | | | | | | |  | | | |  | |  |
|  | |  | | | | DIDN’T THINK IT WAS NECESSARY 7 | | | | | | | | |  | | | |  | |  |
| 10506 | | Has a condom broke or failed anytime in the last 30 days with any paying client? | | | | YES 1 | | | | | | | | |  | | | |  | |  |
|  | |  |  |  |  | NO 2  DON’T REMEMBER 7 | | | | | | | | |  | | | |  | |  |
|  | |  |  |  |  |  | | | | | | | | |  | | | |  | |  |
| 10507 | | With how many different paying partners did you have sex without condoms in the last 30 days? | | | |  | | | | | | | | |  | | | |  | |  |
|  | |  |  |  |  | PP NO COND N \|__\|__\| | | | | | | | | |  | | | |  | |  |
|  | |  |  |  |  |  | | | | | | | | |  | | | |  | |  |
|  | | It is common for some clients to ask for anal sex that means man inserts his penis in your anus.  Have you ever had anal sex with a client? When? | | | | YES, IN THE LAST 30 DAYS 1  YES, BEFORE LAST 30 DAYS 2 | | | | | | | | |  | | | |  | |  |
| 10508 | |  |  |  |  | NEVER 3 | | | | | | | | | 10601 | | | |  | |  |
|  | |  |  |  |  | REFUSED TO RESPOND 9 | | | | | | | | | 10601 | | | |  | |  |
| 10509 | | Did you use condom the last time you had anal sex? | | | | YES 1 | | | | | | | | |  | | | |  | |  |
|  | |  |  |  |  | NO 2 | | | | | | | | |  | | | |  | |  |
|  | |  | | | |  | | | | | | | | |  | | | |  | |  |
| **SECTION 6. EXPOSURE TO SEXUAL RISK: NON-PAYING PARTNERS** | | | | | | | | | | | | | | | | | | |  | |  |
|  | | **Now I want to talk to you about partners who do not usually pay for sex.** | | | | | | | | | | | | |  | | | |  | |  |
| 10601 | | How many different sexual partners have you had in the last 30 days who did not pay for sex? | | | NP VPART \|__\|__\| | | | | | | | | | |  | | | |  | |  |
|  | |  |  |  | NONE 00 | | | | | | | | | | 10603 | | | |  | |  |
|  | |  | | |  |  |  |  |  |  |  |  |  |  |  |  |  |  |  | |  |
| 10602 | | What is your relationship to the most recent non-paying partner? | | | SPOUSE 1 | | | | | | | | | | 10604 | | | |  | |  |
|  | |  |  |  | OTHER REGULAR PARTNER 2 | | | | | | | | | |  | | | |  | |  |
|  | |  |  |  | ONE-TIME PARTNER 3 | | | | | | | | | |  | | | |  | |  |
| 10603a | | Do you have a current regular sexual partner? | | | Yes 1  No 2 | | | | | | | | | | 10701 | | | |  | |  |
|  | |  | | |  | | | | | | | | | |  | | | |  | |  |
|  | |  | | |  | | | | | | | | | |  | | | |  | |  |
| 10603b | | How long have you been having sexual relations with the most recent regular partner? | | | **UNIT** | | | | | | | | | |  | | | |  | |  |
|  | |  |  |  | DAYS 1 | | | | | | | | | | NUMBER \|__\|__\| | | | |  | |  |
|  | |  |  |  | MONTHS 2 | | | | | | | | | |  | | | |  | |  |
|  | | ***CIRCLE UNIT AND ENTER NUMBER*** | | | YEARS 3 | | | | | | | | | |  | | | |  | |  |
| 10604 | | Do you normally live together with this person? | | | YES 1 | | | | | | | | | |  | | | |  | |  |
|  | |  |  |  | NO 2 | | | | | | | | | |  | | | |  | |  |
|  | | What is your regular non-paying partner occupation? | | | UNEMPLOYED 0 | | | | | | | | | |  | | | |  | |  |
| 10605 | |  |  |  | CIVIL SERVANT 1 | | | | | | | | | |  | | | |  | |  |
|  | |  |  |  | TRANSPORT WORKER 2 | | | | | | | | | |  | | | |  | |  |
|  | |  | | | CONSTRUCTION WORKER/DAILY LABORER 3 | | | | | | | | | |  | | | |  | |  |
|  | |  | | | STUDENT 4 | | | | | | | | | |  | | | |  | |  |
|  | |  | | | FARMER 5 | | | | | | | | | |  | | | |  | |  |
|  | |  | | | UNIFORMED SERVICE 6 | | | | | | | | | |  | | | |  | |  |
|  | |  | | | BUSINESS/TRADE/RETAIL 7  OTHERS DON’T MENTIONED ABOVE 8 | | | | | | | | | |  | | | |  | |  |
|  | |  | | | DON'T KNOW 9 | | | | | | | | | |  | | | |  | |  |
| 10606 | | Did you use a condom during the last sexual intercourse with him? | | | YES 1  NO 2  DON’T REMEMBER 7 | | | | | | | | | |  | | | |  | |  |
|  | |  |  |  |  | | | | | | | | | |  | | | |  | |  |
| 10607 | | When have you had your last anal sex experience with a non- paying partner? | | | YES WITHIN THE LAST12 MONTHS 1 | | | | | | | | | |  | | | |  | |  |
|  | |  |  |  | YES BEFORE THE LAST 12 MONTHS 2 | | | | | | | | | | 10701 | | | |  | |  |
|  | |  |  |  | NEVER 3 | | | | | | | | | | 10701 | | | |  | |  |
|  | |  |  |  | REFUSED TO ANSWER 9 | | | | | | | | | | 10701 | | | |  | |  |
| 10608 | | How many times did you have anal sex with non-paying partners with in the last 3 months? | | |  | | | | | | | | | |  | | | |  | |  |
|  | |  |  |  | NP ASEX \|__\|__\|__\|  if no 00 | | | | | | | | | |  | | | |  | |  |
|  | |  |  |  |  | | | | | | | | | |  | | | |  | |  |
| 10609 | | Do you use condoms last time you had anal sex (with non-paying partners)? | | | YES 1 | | | | | | | | | |  | | | |  | |  |
|  | |  |  |  | NO 2 | | | | | | | | | |  | | | |  | |  |
|  | |  | | |  | | | | | | | | | |  | | | |  | |  |
|  | |  | | |  | | | | | | | | | |  | | | |  | |  |
| **SECTION 7. ALCOHOL AND KHAT** | | | | | | | | | | | | | | |  | | | |  | |  |
| **Q.N** | | **Questions** | | | | | **Coding & response categories** | | | | | | | | **Skip to** | | | |  | |  |
| 10701 | | How often do you have a drink containing alcohol? | | | | | NEVER 0 | | | | | | | | 10705 | | | |  | |  |
|  |  |  |  |  |  |  | ONCE A MONTH OR LESS 1 | | | | | | | |  | | | |  | |  |
|  |  |  |  |  |  |  | 2 – 4 TIMES A MONTH 2 | | | | | | | |  | | | |  | |  |
|  |  |  |  |  |  |  | 2 – 3 TIMES A WEEK 3 | | | | | | | |  | | | |  | |  |
|  |  |  |  |  |  |  | 4 OR MORE A WEEK 4 | | | | | | | |  | | | |  | |  |
| 10702 | | How many standard drinks containing alcohol do you have on typical day?  **See the given picture** | | | | | 1 or 2 0  3 or 4 1  5 or 6 2  7 to 9 3  10 or more 4 | | | | | | | |  | | | |  | |  |
| 10703 | | How often do you have 6 or more drinks on one occasion? | | | | | NEVER 0  LESS THAN ONCE A MONTH 1  MONTHLY 2  WEEKELY 3  DAILY OR ALMOST DAILY 4 | | | | | | | |  | | | |  | |  |
| 10704 | | In the last 30 days have you ever drunk so much that you can’t remember what happened the next day? | | | | | YES, IN LAST 30 DAYS 1 | | | | | | | |  | | | |  | |  |
|  | |  |  |  |  |  | YES, BEFORE LAST 30 DAYS 2 | | | | | | | |  | | | |  | |  |
|  | |  |  |  |  |  | NO 3 | | | | | | | |  | | | |  | |  |
|  | |  |  |  |  |  | DON’T REMEMBER 7 | | | | | | | |  | | | |  | |  |
| 10705 | | How many days in the week do you normally chew khat? | | | | | 5-7 DAYS PER WEEK 1 | | | | | | | |  | | | |  | |  |
|  | |  |  |  |  |  | 3-4 DAYS PER WEEK 2 | | | | | | | |  | | | |  | |  |
|  | |  |  |  |  |  | 1-2 DAYS PER WEEK 3 | | | | | | | |  | | | |  | |  |
|  | |  |  |  |  |  | LESS THAN ONCE A WEEK 4 | | | | | | | |  | | | |  | |  |
|  | |  |  |  |  |  | NEVER 5 | | | | | | | |  | | | |  | |  |
| 10706 | | Some people use other drugs besides alcohol and khat for pleasure. Some are chewing, some are smoked, and some are injected with a needle.  What type drugs have you used in the last 30 days?  CIRCLE ALL MENTIONED | | | | | I HAVE NEVER USED ANYDRUGS 1  CHEWING DRUGS 2 | | | | | | | | Skip to next section | | | |  | |  |
|  | |  |  |  |  |  | SMOKED DRUGS 3 | | | | | | | |  | | | |  | |  |
|  | |  |  |  |  |  | OTHER INGESTABLE 4 | | | | | | | |  | | | |  | |  |
|  | |  |  |  |  |  | OTHER INJECTATABLE 5  OTHER 6 | | | | | | | |  | | | |  | |  |
|  | |  |  |  |  |  |  | | | | | | | |  | | | |  | |  |
|  | |  | | | | |  | | | | | | | |  | | | |  | |  |

| **SECTION 8. STI** | | |  |  |
| --- | --- | --- | --- | --- |
| **Q.N** | **Questions** | **Coding & response categories** | **Skip to** |  |
| 10801 | Have you had any unusual **vaginal discharge** in the past 12 months? | YES 1 |  |  |
|  |  | NO 2 |  |  |
|  |  | DON’T KNOW 8 |  |  |
|  |  | REFUSED TO ANSWER 9 |  |  |
| 10802 | Have you had a **genital ulcer** in the past 12 months? | YES 1 |  |  |
|  |  | NO 2 |  |  |
|  |  | DON’T KNOW 8 |  |  |
|  |  | REFUSED TO ANSWER 9 |  |  |
|  | **Check if the above Q10801 – 10802 any of symptom reported** | Yes 1  No 2 | Skip if no to 10806 |  |
|  |  |  |  |  |
| 10803 | Where did you go first for treatment the last time you had any of these symptoms? | PUBLIC HF 1 |  |  |
|  |  | PRIVATE HF 2 |  |  |
|  |  | PHARMACY 3 |  |  |
|  |  | HOME REMEDY, TRADITIONAL or SELF TREATMENT 4 |  |  |
|  |  | STI CONFIDENTAL CLINIC 5 |  |  |
|  |  | I DID’T SEEK TREATMENT 6 | 10806 |  |
|  |  | DON’T/CAN’T REMEMBER 7 | 10806 |  |
|  |  |  |  |  |
| 10804 | What kind of treatment did you receive for your most recent problem? CIRCLE AL MENTIONED | ADDIS CURE KIT 1 |  |  |
|  |  | PILLS 2 |  |  |
|  |  | INJECTION 3 |  |  |
|  |  | HERBAL REMEDIES 4 |  |  |
| 10805 | Have you ever experienced any dishonor/ discrimination from health care providers as a result of selling sex? Which health care providers? | NO DISCRIMINATION 1 |  |  |
|  |  | IN PUBLIC HF 2 |  |  |
|  |  | IN PRIVATE HF 3 |  |  |
|  |  | IN PHARMACY 4 |  |  |
|  |  | IN TRADITIONAL/HERBALIST 5 |  |  |
|  |  | DON’T REMEMBER 7 |  |  |
|  |  |  |  |  |
| 10806 | For each of the following groups, say whether you feel accepted, neutral, or rejected by most people because of the work that you do? | 1=Accepted, 2=Neutral, 3= Rejected, 4=D k |  |  |
|  |  | LOCAL POLICE 1 2 3 4 |  |  |
|  |  | HEALTH WORKERS 1 2 3 4 |  |  |
|  |  | WORK MATES 1 2 3 4 |  |  |
|  |  | OTHER COMMUNITY MEMBERS 1 2 3 4 |  |  |
|  |  | FAMILY AT HOME 1 2 3 4 |  |  |

|  | | |  | |  | | |  |  |
| --- | --- | --- | --- | --- | --- | --- | --- | --- | --- |
| **SECTION 9. SEXUAL VIOLENCE** | | | | | | | |  |  |
| **Q.N** | **Questions** | | | | | | **Coding & response categories** | **Skip to** |  |
|  | ***Now I’d like to ask you some questions about sexual violence and rape. With rape we mean when a man either forces or threatens to hurt you to have vaginal or anal sex against your will.*** | | | | | | |  |  |
| 10901 | Have you ever been physically beaten by a sexual partner or client in the last 12 months? | | | | | | \| NEVER 1 \| \| --- \| |  |  |
|  |  |  |  |  |  |  | YES, BY A PAYING PARTNER 2 |  |  |
|  |  |  |  |  |  |  | \| YES, BY NON PAYING PARTNERYES 3 \| \| --- \| |  |  |
|  |  |  |  |  |  |  | BY BOTH 4 |  |  |
|  |  | | | | | | REFUSED TO ANSWER 9 |  |  |
| 10902 | Have you ever been raped or forced to have sex against your will since you start selling sex? By who raped most recently? | | | | | | NO 0  BY PAYING CLIENT 1 | 11001  11001 |  |
|  |  | | | | | | BY NON-PAYING PARTNER 2 |  |  |
|  |  | | | | | | BY EMPLOYER 3 |  |  |
|  |  | | | | | | BY POLICEMAN 4 |  |  |
|  |  | | | | | | MIDDLE MAN 5 |  |  |
|  |  | | | | | | SOMEONE YOU DIDN’T KNOW 6  OTHERS 7  REFUSED TO RESPOND 9 |  |  |
|  |  | | | | | |  |  |  |
| 10903 | The last time you were raped did you report to the police? | | | | | | YES 1 |  |  |
|  |  |  |  |  |  |  | NO 2 |  |  |
|  |  |  |  |  |  |  | REFUSED TO ANSWER 9 |  |  |
| 10904 | The last time you were raped did you seek health care/treatment? | | | | | | YES 1 |  |  |
|  |  |  |  |  |  |  | NO 2 |  |  |
|  |  |  |  |  |  |  | REFUSED TO ANSWER 9 |  |  |
|  |  | | | | | |  | | |
|  |  | | | | | |  | | |
| **SECTION10. KNOWLEDGE AND ATTITUDES TOWARDS HIV/AIDS** | | | | | | | | | |
| **Q.N** | **Questions** | | | | | **Coding & response categories** | | **Skip to** |  |
| 11001 | Please indicate the main ways a person can protect her/himself against HIV infection? | | | | | ABSTAINING 1 | |  |  |
|  |  |  |  |  |  | BEING FAITHFUL TO ONE PARTNER 2 | |  |  |
|  |  |  |  |  |  | PROPER AND CONSISTENT CONDOM USE 3 | |  |  |
|  |  |  |  |  |  | NOT SHARING NEEDLES/SHARPS 4 | |  |  |
|  |  | | | | | HAVE PARTNER GET TESTED 5  AVOIDING CONTACT WITH BODY FLUID OF INFECTED 6 | |  |  |
|  | ***PROBE:* Any other? *CIRCLE ALL MENTIONED*** | | | | | MISCONCEPTIONS7 | |  |  |
|  |  | | | | |  | |  |  |
| 11002 | Can the virus that causes AIDS be transmitted from a mother to her baby during pregnancy? During delivery? During breast feeding**? Circle all answered** | | | | | DURING PREGNANCY1 | |  |  |
|  |  |  |  |  |  | DURING DELIVERY 2 | |  |  |
|  |  |  |  |  |  | DURING BREAST FEEDING 3 | |  |  |
|  |  |  |  |  |  |  | |  |  |
|  | ***Do you mostly agree or disagree with the following statements:*** | | | | | **Interviewer: be sure to read the questions in a non-biased way (do not make it clear which is the “right” answer)** | |  |  |
| 11003 | “*I am not as careful about HIV and sex now because there is better treatment for AIDS*” | | | | | AGREE 1 | |  |  |
|  |  |  |  |  |  | DISAGREE 2 | |  |  |
|  |  |  |  |  |  | UNSURE 7 | |  |  |
| 11004 | *Once you have unprotected sex with someone, there is no reason to use condoms again to prevent HIV with that person.* | | | | | AGREE 1 | |  |  |
|  |  |  |  |  |  | DISAGREE 2 | |  |  |
|  |  |  |  |  |  | UNSURE 7 | |  |  |
|  |  | | | | |  | |  |  |
|  |  | | | | |  | |  |  |
| **SECTION 11. HIV COUNSELLING AND TESTING** | | | | | | | |  |  |
| **Q.N** | | **Questions and filters** | | **Coding & response categories** | | | | **Skip to** |  |
| 11101 | | HOW LONG AGO WERE YOU LAST TESTED  and received for HIV? | | MONTHs \|__\|__\|__\| | | | |  |  |
|  | |  | | Never Tested 000 | | | | 11201 |  |
|  | |  | | DON’T REMEMBER 997 | | | |  |  |
|  | |  | | REFUSED TO ANSWER 999 | | | | 11201 |  |
| 11102 | | How many times have you tested your HIV status in the last 12 Months? | | \|___\|___\| | | | |  |  |
|  | |  |  | DON’T REMEMBER 97 | | | |  |  |
|  | |  |  | REFUSED TO ANSWER 99 | | | |  |  |
|  | |  | |  | | | |  |  |
| 11103 | | Where did you test for HIV most recently? | | PUBLIC HF1 | | | |  |  |
|  | |  |  | PRIVATE HF 2 | | | |  |  |
|  | |  |  | NGO HF 3 | | | |  |  |
|  | |  | | CONFIDENTIAL STI CLINIC 4 | | | |  |  |
|  | |  | | MOBILE VCT SERVICE 5 | | | |  |  |
|  | |  | | HEWS/HEALTH POST 6  DON’T KNOW 8  REFUSED TO ANSWER 9 | | | |  |  |
| 11104 | | Has your current regular partner ever shared his HIV results with you? | | YES 1  NO 2  NO REGULAR PARTNER 3 | | | | 11106  11106 |  |
| 11105 | | As far as you know, has your current partner ever tested positive for HIV’? | | YES 1  NO 2 | | | |  |  |
| **The next questions are important for knowing the quality of care you received.** | | | | | | | |  |  |
| 11106 | | Have you ever tested positive for the HIV virus? | | YES 1 | | | |  |  |
|  | |  |  | NO 2 | | | | 11201 |  |
|  | |  |  | REFUSED TO ANSWER 9 | | | | 11201 |  |
| 11107 | | Have you disclosed your status to your current regular non-paying partner? | | YES 1 | | | |  |  |
|  | |  |  | NO 2  I DON’T HAVE NON-PAYING PARTNER 3 | | | |  |  |
|  | |  |  | DON’T REMEMBER 8 | | | |  |  |
|  | |  | | REFUSED TO ANSWER 9 | | | |  |  |
| 11108 | | How long ago did you first test HIV positive? | | MONTHs \|__\|\|__\|__\|  IF LESS THAN ONE MONTH 000 | | | |  |  |
|  | |  |  | DON’T REMEMBER 997 | | | |  |  |
|  | |  |  | REFUSED TO ANSWER 999 | | | |  |  |
| 11109 | | Have you ever attended a support group for HIV positive people? In the last 12 months? | | YES, IN LAST 12 MONTHS 1 | | | |  |  |
|  | |  |  | YES, BUT NOT IN LAST 12 MONTHS 2 | | | |  |  |
|  | |  |  | NEVER 3 | | | |  |  |
|  | |  | |  | | | |  |  |
| 11110 | | Are you currently taking any medication/treatment regularly?  **Select all that apply** | | NEVER 1 | | | | 11201 |  |
|  | |  |  | CPT (Co-Trimoxazole) 2 | | | | 11201 |  |
|  | |  |  | ART 3 | | | |  |  |
|  | |  |  | PMTCT DRUGS 4 | | | | 11201 |  |
|  | |  | | HERBAL 5 | | | | 11201 |  |
|  | |  | | HOLY WATER 6 | | | | 11201 |  |
|  | |  | | REFUSED TO ANSWER 9 | | | | 11201 |  |
| 11111 | | If ART checked, In what month and year did you start your treatment with ART?  **(Ethiopian)** | |  | | | |  |  |
|  | |  |  | MONTH \|__\|__\| | | | |  |  |
|  | |  |  | UNSURE 97 | | | |  |  |
|  | |  |  | REFUSED TO ANSWER 99 | | | |  |  |
|  | |  | | YEAR \|__\|__\|__\|__\| | | | |  |  |
|  | |  | | UNSURE 9997 | | | |  |  |
|  | |  | | REFUSED TO ANSWER 9999 | | | |  |  |
|  | |  | |  | | | |  |  |
| **SECTION 12. PREVENTION PROGRAM EXPOSURE** | | | | | | | |  |  |
| **Q.N** | | | **Questions** | **Coding & response categories** | | | | **Skip to** |  |
| 11201 | | | Where do you usually get condoms? | PUBLIC HF 1 | | | |  |  |
|  | | |  | PRIVATE HF 2 | | | |  |  |
|  | | | ***PROBE*: Any place else?** | NGO 3 | | | |  |  |
|  | | | **CIRCLE ALL MENTIONED** | BARS/HOTELS/ENTERTAINMENTS 4 | | | |  |  |
|  | | |  | SHOPS/KIOSKS 5 | | | |  |  |
|  | | |  | HEALTH EXTENSION WORKERS/HEALTHPOST 6 | | | |  |  |
|  | | |  | PEER OUTREACH 7 | | | |  |  |
|  | | |  | PROMOTIONAL EVENTS 8 | | | |  |  |
|  | | |  | PARTNERS 9 | | | |  |  |
| 11202 | | | In the last 30 days have you ever had a sex without condom because you didn’t find one? | YES 1 | | | |  |  |
|  | | |  | NO 2 | | | |  |  |
|  | | |  | DON’T REMEMBER 7 | | | |  |  |
| 11203 | | | Have you ever been part of a guided peer group discussion concerning HIV? In the last 12 months? | YES, IN LAST 12 MONTHS1 | | | |  |  |
|  | | |  | YES, BEFORE LAST 12 MONTHS2 | | | |  |  |
|  | | |  | NOT AT ALL 3 | | | |  |  |
|  | | |  | I DON’T REMEMBER 7 | | | |  |  |
| 11204 | | | Are you currently a member of any association related to sex work? | YES 1 | | | |  |  |
|  | | |  | NO 2 | | | |  |  |
|  | | |  | NOT AWAR E OF THE PRESENS OF THE ASSOCIATION 3 | | | |  |  |
| 11205 | | | In the last 12 months, have you ever called into the AIDS hotline (952)? | YES 1 | | | |  |  |
|  | | |  | NO 2 | | | |  |  |
| 11206 | | | Have you ever been a part of any Income Generation Activity? In the last 12 months? | YES, IN LAST YEAR 1  YES, BEFORE LAST YEAR 2  NO 3  UNSURE 7  REFUSED TO ANSWER 9 | | | |  |  |
|  | | |  |  | | | |  |  |
|  | | |  |  | | | |  |  |
|  | | | ***THANK YOU!*** | ***Finished System time ----- : ------*** | | | | |  |
|  | | |  |  | | | |  |  |
|  | | |  |  | | | |  |  |
|  | | |  |  | | | |  |  |
|  | | |  |  | | | |  |  |
|  | | |  |  | | | |  |  |
|  | | |  |  | | | |  |  |
